# HOXA10 DNA Methylation Level in the Endometrium Women with Endometriosis: A Systematic Review

**DOI:** 10.3390/biology12030474

**Published:** 2023-03-20

**Authors:** Marjanu Hikmah Elias, Nurunnajah Lazim, Zulazmi Sutaji, Mohammad Azrai Abu, Abdul Kadir Abdul Karim, Azizah Ugusman, Saiful Effendi Syafruddin, Mohd Helmy Mokhtar, Mohd Faizal Ahmad

**Affiliations:** 1 Advanced Reproductive Centre (ARC) HCTM UKM, Department of Obstetrics & Gynecology, Faculty of Medicine, National University of Malaysia, Jalan Yaacob Latiff, Bandar Tun Razak, Kuala Lumpur 56000, Malaysia; 2 Faculty of Medicine & Health Sciences, Universiti Sains Islam Malaysia, Nilai 71800, Negeri Sembilan, Malaysia; 3 Department of Physiology, Faculty of Medicine, National Univeristy of Malaysia, Jalan Yaacob Latiff, Bandar Tun Razak, Kuala Lumpur 56000, Malaysia; 4 Medical Molecular Biology Institute, National University of Malaysia, Jalan Yaacob Latiff, Bandar Tun Razak, Kuala Lumpur 56000, Malaysia

**Keywords:** DNA methylation, endometriosis, endometrium, eutopic endometrium, ectopic endometrium, secretory phase, proliferative phase

## Abstract

**Simple Summary:**

Endometriosis is an inflammatory chronic systemic disease resulting in pelvic pain and infertility. However, despite a high prevalence of endometriosis, disease identification is still insufficient, and a high percentage of misdiagnosing was observed. Our primary interest is mainly the aberrant hypermethylation of HOXA10 has been reported to play a role in endometriosis. Hence, a comprehensive literature search to identify the DNA methylation level of HOXA10 among endometriosis patients across populations was performed. Our gathered evidence was classified as high-quality studies, and we found a higher HOXA10 DNA methylation level in the endometrium tissue of women with endometriosis in all the included studies. Our findings can help share the light of HOXA 10 methylation among women with endometriosis.

**Abstract:**

Endometriosis is an inflammatory chronic systemic disease resulting in pelvic pain and infertility. However, despite a high prevalence of endometriosis, disease identification is still insufficient, and a high percentage of misdiagnosing was observed. Hence, a comprehensive study needs to be done to improve our understanding of the pathogenesis of endometriosis. Aberrant hypermethylation of HOXA10 has been reported to play a role in endometriosis. Thus, a comprehensive literature search was conducted to identify the DNA methylation level of HOXA10 among endometriosis patients across populations. The literature search was done using PubMed, Scopus, EBSCOhost, and Science Direct applying (HOXA10 OR “homeobox A10” OR “HOXA-10” OR HOX1) AND (“DNA methylation” OR methylation) AND (endometriosis OR endometrioma) as keywords. From 491 retrieved studies, five original articles investigating the DNA methylation level of HOXA10 from endometrium tissues among endometriosis women were included. All five included studies were classified as high-quality studies. High HOXA10 DNA methylation level was observed in the endometrium tissue of women with endometriosis in all the included studies. The secretory phase was identified as the best sampling time for HOXA10 DNA methylation study in endometriosis, and the most studied DNA methylation site is the promoter region of the HOXA10. However, more studies are needed to expose the HOXA10 mechanism in the pathogenesis of endometriosis.

## 1. Introduction

Endometriosis is a chronic systemic disease associated with pelvic pain and infertility [1], with 84% experiencing symptoms before diagnosis [2]. This disease has not only physical effects but also psychological ones by causing depression, anxiety, and compromising sexual life, work, and social relationships [3]. Endometriosis is distinguished by infiltration into the muscularis propria of hollow viscera or infiltrating lesions underneath the peritoneum [4]. Endometriosis affects the endometrium tissue in the uterus (eutopic endometrium) and outside of the uterus, leading to ectopic endometrium growth. Endometrioid-type glands and stroma, usually associated with pigment-laden macrophages, are the histological results in endometriosis tissue [5]. However, the absence of visible lesions or negative histology should not exclude the diagnosis of endometriosis, as occult endometriosis has been documented [1]. Therefore, despite this high prevalence of endometriosis, disease identification still needs to be improved. Furthermore, it takes up to 11 years to diagnose, with 65% of the women being misdiagnosed at first [6]. Hence, there is a need for molecular biomarkers to be implemented for the diagnosis and prognosis of endometriosis to expedite the time of diagnosis to allow the most appropriate treatment to be given to the patients as early as possible. 

Several theories have been proposed for the pathogenesis and etiology of endometriosis. The theories include the Sampson hypothesis, which proposed that retrograde menstruation allows implantation of the endometrial gland and stroma into the peritoneal cavity, supported by several studies [7]. In comparison, coelomic metaplasia theory suggested that the metaplastic phenomenon could cause endometriosis to develop in all celomic wall derivatives [8]. Lymphatic or hematogenous vessels facilitated the dissemination of endometrial cells to other organs and tissues [9]. At different anatomical sites, endometrial cells could act like hematopoietic stem cells and differentiate into endometriotic tissue [10]. Thus, the molecular effects of these theories are crucial to be investigated. One of the possible genes affected by these theories is HOXA10.

HOXA10 is a transcription factor involved in cell proliferation and apoptosis of various cancers [11,12,13]. HOXA10 is located at chromosome 7, specifically at p15.2 (7p15.2), containing 3 protein-coding exons. Among the tissues in the body, HOXA10 is highly expressed in the endometrium and prostate tissues. However, HOXA10 expression is lacking in the ovary, testes, stomach, lungs, and liver. The expression of HOXA10 in a specific tissue has been associated with several diseases, including endometrial cancer [14,15], prostate cancer [16], acute myeloid leukemia [14], gastric cancer [17], and many other cancers. In endometriosis, HOXA10 has been proposed as one of the potential biomarkers for the diagnosis of endometriosis [18]. The loss of HOXA10 expression has been reported to cause endometrial hyperplasia leading to endometrial cancer [15]. Aberrant hypermethylation of HOXA10 has been reported to play a role in endometriosis [19] and implantation failures in women receiving in vitro fertilization (IVF) treatment [20]. In the endometrium, HOXA10 expression in glandular and stromal cells of the endometrium is regulated by steroid hormones [21], making the DNA methylation level and HOXA10 expression fluctuate between menstrual cycles. Hence, the best time for sample collection should be identified and standardized to eliminate the confounding factor influencing the DNA methylation level. Another knowledge gap is also seen in the regulation of HOXA10 expression studies in endometriosis. The expression of HOXA10 can be regulated through various factors including epigenetic factor such as hypermethylation.

The epigenetic changes usually vary between populations, influenced by environmental factors [9]. However, there is still a need to report the variation in the HOXA10 DNA methylation level among endometriosis patients across populations. In addition, the DNA methylation site in the HOXA10 gene that has been studied and published in the literature has yet to be assembled and pictured clearly. Thus, this systematic review mainly aims to identify the DNA methylation level of HOXA10 across populations. This study also aims to identify the best sampling time for the HOXA10 DNA methylation study and to assemble the DNA methylation sites in the HOXA10 gene that have been studied and published in the literature.

## 2. Materials and Methods

A systematic review of the publications was completed following the PRISMA guideline to identify studies that presented the HOXA10 DNA methylation level in the endometrium of endometriosis patients.

### 2.1. Search Strategy

Science Direct, Scopus, EBSCOhost and PubMed databases were searched to identify relevant studies with unlimited starting publication dates until 1 August 2022. Synonyms for keywords were developed through MeSH terms from the Cochrane Library, and further terms were discovered by evaluating assembled review articles. For example, the following combination of keywords was searched: (HOXA10 OR “homeobox A10” OR “HOXA-10” OR HOX1) AND (“DNA methylation” OR methylation) AND (endometriosis OR endometrioma). Other references were determined from the bibliographies of the retrieved literature.

### 2.2. Inclusion Criteria

Case-control and cross-sectional studies with abstracts investigating the DNA methylation level of the HOXA10 gene among endometriosis women were included. Only clinical studies that use endometrial tissues as their sample were included to ensure data homogeneity.

### 2.3. Exclusion Criteria

Narrative reviews, editorials, case reports, and conference proceedings were excluded from in vitro, in vivo, and in silico studies. Studies involving responses toward hormonal treatment or intervention studies, such as using an intrauterine device (IUD), are also excluded. Studies utilizing blood or tissues other than endometrium as their sample were excluded. These criteria were employed to accomplish this systematic review’s objective of confining the typical methylation level in endometriosis women.

### 2.4. Study Selection

All recovered articles were filtered in three phases. In the first phase, duplicate articles were excluded by using EndNote X9 software. The title and abstract of the remaining articles were screened in the second phase. All full texts were retrieved and screened in the third phase. Finally, all articles that did not meet the inclusion and exclusion criteria were excluded. MHE and MFA made the selection of eligible studies. Disagreements about inclusion were resolved by consensus.

### 2.5. Data Extraction and Study Quality

Data extraction was done independently by MHE and MFA. The data were extracted into a predefined Excel spreadsheet. The information extracted includes the first name of the last author (as the article ID), study design, sample size, type of sample collected, time of sample collection, the method used to measure methylation level, and DNA methylation results. The DNA methylation site analyzed by all the selected studies was also extracted and aligned using BioEdit Software. The quality of included studies was evaluated unassisted by MHE and MFA. Joanna Briggs Institute (JBI) critical appraisal tools were used for the study quality assessment [22]. The studies were categorized as low-quality studies (high-risk of bias) if the overall score was less than 50%.

## 3. Results

### 3.1. Study Selection

**Figure 1:**
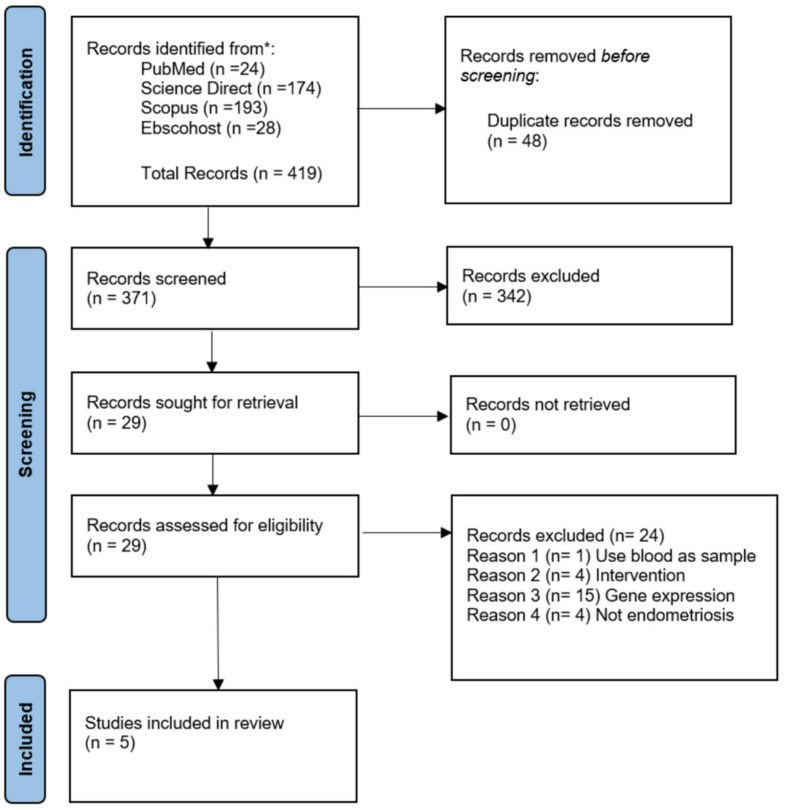
PRISMA flow diagram for studies selection in this systematic review.

From the keywords used, the initial search identified 419 studies. After removing 48 duplicates, the title and abstracts of 371 studies were screened. A total of 342 studies were excluded as those did not fulfill the objectives of this systematic review. In the third phase, 29 full texts were screened, and 24 studies were excluded. The reason for exclusion is mentioned in Figure 1. Thus, five studies were included in this systematic review (Figure 1). 

### 3.2. Study Characteristic

The characteristics and references of the included studies are shown in Table 1. The studies included in this systematic review data are from between 2005 and 2019. One hundred eighty-one women from five studies were included in this systematic review. The method for detecting or measuring HOXA10 DNA methylation level varies from Chromatin Immunoprecipitation Real-Time PCR, DNA methylation array, Methylation Specific PCR, Pyrosequencing, and Bisulfite Sequencing. Thus, the methylation results vary in only reporting the overall methylation level of the amplified DNA or the methylation level of each CpG site in the amplified DNA.

### 3.3. HOXA10 DNA Methylation

The mean percentage of DNA methylation level was identified from all the included studies and summarised in Table 2. All case-control studies compared the HOXA10 DNA methylation level between endometriosis and normal women. The HOXA10 DNA methylation level was significantly higher in the endometrial tissue of the women with endometriosis. The same pattern of DNA methylation was observed in all the included studies with different populations. Three studies (Samadieh et al. (2019), Ji et al. (2017), and Andersson et al. (2014)) compared the HOXA10 expression level between eutopic and ectopic among endometriosis patients. The HOXA10 DNA methylation level was significantly higher in the eutopic endometrial tissue, which corresponded to ectopic endometrial tissue during the secretory phase. The methylation percentage for Samadieh et al. (2019) was taken from the bar chart included in the article. The overall mean DNA methylation percentage for Wu et al. (2005) was calculated based on the bisulfite sequencing results of fragments F1, F2, and F3.

**Figure 2:**
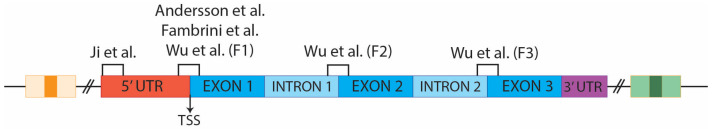
The position of the DNA methylation in HOXA10 analyzed by the included studies [23,24,25,26].

The sites of DNA methylation analyzed by all included studies were extracted and aligned. Ji et al. analyzed the DNA methylation at the 5′untranslated region (5′UTR), far from the transcription start site (TSS). Samadieh et al. (2019), Anderson et al. (2014), Fambrini et al. (2013), and Wu et al. (2005) (F1) analyzed the DNA methylation at approximately the same region starting at the end of 5′UTR, crossing the TSS and ending at the beginning of exon 1. While Wu et al. (2005) also analyze the DNA methylation from the end of intron one until the beginning of exon 2 (F2) and from the end of intron two until exon three (F3). Figure 2 and Table 3 show the position of all the sites analyzed in HOXA10 by all studies.

### 3.4. Study Quality

A detailed quality assessment of the incorporated studies is included in Table A1. The analysis revealed that 100% of the incorporated studies were high-quality (low risk of bias). Thus, all studies were included in this systematic review.

## 4. Discussion

DNA methylation is the most common epigenetic modification in the hormonal and immunological aberrations in the endometrium [27,28]. In all case-control studies included in this systematic review, the methylation level of HOXA10 in eutopic endometrium collected during the secretory phase is significantly higher in endometriosis patients compared to normal individuals. However, the HOXA10 DNA methylation level in ectopic endometrium was not significantly different from normal endometrium. Thus, it is estimated that the HOXA10 expression level was downregulated in the eutopic endometrium but was upregulated in the ectopic endometrium of endometriosis patients. These statements are supported by Mirabutalebi et al. (2018), that found a significant decrease in HOXA10 expression in eutopic endometrium but not in ectopic endometrium [29]. These findings of HOXA10 DNA methylation are similar across populations. Thus, indicating that the variations in geography and population are not the confounding factors for HOXA10 DNA methylation in endometriosis patients.

HOXA10 is a transcription factor that plays a crucial role in the functional differentiation of the endometrium [18]. HOXA10 is highly expressed in the endometrium’s luminal epithelium, glandular epithelium, and stroma cells. Therefore, in endometriosis, the downregulation of HOXA10 through DNA methylation could alter the uterine environment, such as reducing the number of pinopods in the eutopic endometrium [30]. Although the mechanism of how HOXA10 impacts endometrial cells is still not precise, there is a study that correlates the expression of HOXA10 with the level of autophagy [31]. The down-regulation of HOXA10 triggers an autophagic function in endometriosis via the Wnt/b-catenin axis [32], b3-integrin [33], FK506 binding protein 4 [34], beclin-I, and LC3-II [35].

While in ectopic endometrium, Samadieh et al. (2019), Ji et al. (2017), and Andersson et al. (2014) reported a low DNA methylation level of HOXA10, reflecting the high expression of HOXA10. Thus, the high level of HOXA10 could be due to the strong expression of stroma surrounding the endometrial gland in the ectopic endometrium [36], mimicking the eutopic endometrium tissue. On the other hand, based on in vitro studies, Kulebyakina et al. (2020) suggested that low expression of HOXA10 in endometrial stromal cells could result in partial loss of HOX unique “code” in endometrial cells, thus assisting ectopy [35]. However, there is still a lack of information on the pathways involved in the pathogenesis of ectopic endometrium. Thus, further functional studies on the pathways related to the effect of HOXA10 in endometriosis are warranted. 

The expression level of HOXA10 in the endometrial tissue changes from time to time, regulated based on the menstrual cyclicity and coordinated by steroid sex hormone level changes [37]. In normal women, HOXA10 expression is low during the proliferative phase but upregulated during the secretory phase [35]. During the secretory phase, high HOXA10 expression is essential to support the cell differentiation and transformation of fibroblast-like endometrial stromal cells into decidual cells in preparation for implantation [38]. However, as reported by all the included studies, the HOXA10 DNA methylation level in endometriosis women increases during the secretory phase. Thus, the low expression of HOXA10 in endometriosis women could inhibit cell differentiation in eutopic endometrium during the secretory phase.

The DNA methylation sites in the HOXA10 also influence the methylation level identified in each study. DNA methylation that occurs in the promoter region of a gene is usually associated with reducing expression [39]. This is because the presence of CpG islands in the promoter is associated with gene regulatory regions. Therefore, the methylation of the CpG island in the promoter region will downregulate the gene expression. Thus, usually, many studies focus on the promoter region for DNA methylation studies. In this systematic review, four studies focus on the promoter region, including some portion of the first exon. The CpG island studied is between -245 bp and 29 bp from the TSS. This region significantly differs in DNA methylation levels between endometriosis and normal women. Apart from endometriosis, the promoter region of HOXA10 also was reported to be methylated in other diseases, such as endometrial cancer [40] and gastric cancer [41].

Furthermore, the high methylation level not only occurred in the promoter region, but Wu et al. also reported on the two other fragments in the first and second intron that are also methylated in women with endometriosis. These findings show that the HOXA10 gene is highly methylated throughout the gene, thus downregulating the HOXA10 expression. Interestingly, low methylation level was reported in women with endometriosis [42], and various treatments used in endometriosis alter HOXA10 regulation [43,44]. Thus, the HOXA10 DNA methylation could be a potential additional biomarker for the diagnosis and prognosis of endometriosis. Apart from DNA methylation, HOXA10 regulation via post-transcriptional modification by miRNA was also reported [29]. However, no further studies were done to confirm the target site of the miRNA on HOXA10, and no functional studies on the effect of miRNA on the HOXA10 expression level were reported. Thus, further functional studies on the effect of DNA methylation on HOXA10 expression are warranted. In addition, the findings from this study could be a door to many other studies, especially on the potential of site-specific DNA demethylation as a targeted therapy for endometriosis.

The identification of HOXA10 DNA methylation level has provided new insight into more potential treatments for endometriosis patients. GnRH agonist and antagonist has been reported to affect the DNA methylation level of the Hoxa10 gene in vivo and consequently affecting uterine receptivity and suppressing the expression of endometrial integrin β3 and finally repressing pinopode growth [45]. Letrozole also has been reported to gain the expression of HOXA10 in endometriosis and improve endometrial receptivity [43]. Furthermore, metformin treatment significantly upregulates the HOXA10 expression and subsequently suppresses the growth of endometriotic cells and potentiates endometrial receptivity [44].

The HOXA10 methylation and expression levels have been identified to play a role in various pathways during the pathogenesis of endometriosis. The pathways include cholesterol synthesis in endometrial stromal cells [21], proliferation and apoptosis [15]. However, more effects from the methylation of HOXA10 should be further studied to understand the pathogenesis of endometriosis robustly. Besides affecting the downstream genes in various pathways, HOXA10 methylation also indirectly affects the pathways by regulating miRNA expression via the crosstalk between epigenetics and microRNA expression. In gastric cancer, the proliferation and invasion of gastric cancer cells are regulated via the promoter hypomethylation and expression of the HOXA10/miR-196b-5p axis [41]. Thus, further studies on the crosstalk between HOXA10 methylation and miRNA expression are needed.

### 4.1. Strengths

The inclusion criteria used ensured only endometrium tissues were collected during the secretory and proliferative phases were implemented to make sure homogenized samples were used, and bias was minimized. In this systematic review, the location of HOXA10 DNA methylation analyzed by each study was re-aligned and mapped. Thus, the site of HOXA10 DNA methylation that are already published are pictured better. To the best of our knowledge, this is the first systematic review that presented all the HOXA10 DNA methylation sites that are already studied.

### 4.2. Limitations

The DNA methylation methods used by the included studies vary. Thus, the depth of DNA methylation level detection between studies is different, which could influence the DNA methylation results and is the significant limitation of this systematic review. All the studies also do not measure the HOXA10 expression level corresponding to the DNA methylation level. Thus, the expression of the HOXA10 in these studies is based on the theory and reports from other studies.

## 5. Conclusions

Based on the systematic review, HOXA10 is methylated in the endometrium of women with endometriosis during the secretory phase, and the methylation pattern is similar across populations. The most studied DNA methylation site of HOXA10 is at the gene’s promoter region. However, additional studies are required to reveal the HOXA10 mechanism in the pathogenesis of endometriosis and to confirm its suitability as a biomarker for the diagnosis and prognosis of endometriosis. Identifying HOXA10 DNA methylation levels in women with endometriosis is also crucial as the base knowledge for developing site-specific DNA demethylation agents in endometriosis treatment.

## Figures and Tables

**Table 1:** Major characteristics of the included studies.

No	Article ID (Reference)	Year	Country	Study Design	Sample size (n)	Type of Sample Collected	Phase of Sample Collection	Method for DNA Methylation
1	Samadieh et al. [19]	2019	Iran	Case-control	36 cases, 21 controls	Eutopic and ectopic endometrial tissue	Proliferative and secretory phase	Chromatin Immunoprecipitation Real-Time PCR Assay
2	Ji et al. [23]	2017	China	Cross-sectional	60	Eutopic and ectopic endometrial tissue	NA	DNA methylation array
3	Andersson et al. [24]	2014	Italy	Case-control	18 cases, 12 controls	Ectopic endometrial tissue	Secretory phase	MSPCR, Pyrosequencing
4	Fambrini et al. [25]	2013	Italy	Case-control	11 cases 11 controls	Endometrial tissue	Secretory phase	Pyrosequencing
5	Wu et al. [26]	2005	USA	Case-control	6 cases 6 controls	Endometrial tissue	Proliferative and early secretory phase	MSPCR, Bisulfite Sequencing.

**Table 2:** Comparison of HOXA10 DNA methylation level between control and endometriosis patients that is divided into types of endometrial tissue sampling.

Article ID	Phase of Menstrual Cycle	HOXA10 DNA Methylation Rate (mean%)		
		Endometriosis		Control
		Eutopic Endometrial Tissue	Ectopic Endometrial Tissue	
Samadieh et al. [19]	proliferative	4	1	4
	secretory	4	0.5	1
Ji et al. [23]	NA	70	35	NA
Andersson et al. [24]	secretory	10.3	5.5	7.75
Fambrini et al. [25]	secretory	13.9		9.7
Wu et al. [26] (F1)	secretory and proliferative	32.5		14.4
(F2)		27		7.3
(F3)		40		2.5

**Table 3:** The DNA methylation locations from the transcription start site analyzed by the included studies.

Article ID	Location from the TSS	Length (bp)
Samadieh et al. [19]	−83–49	113
Ji et al. [23]	−7090–−7018	73
Andersson et al. [24]	−243–18	261
Fambrini et al. [25]	−245–20	265
Wu et al. [26] (F1)	−245–20	265
(F2)	1251–1541	290
(F3)	2026–2241	215

## Data Availability

Not applicable.

## Appendix A

**Table A1:** Quality assessment of the included studies.

No.	Author, Year	Questions Assessing Case-Control Studies										Yes (%)
		1	2	3	4	5	6	7	8	9	10	
1	Samadieh et al., 2019 [19]	Y	Y	Y	Y	Y	Y	Y	Y	Y	Y	100
2	Andersson et al., 2014 [24]	Y	Y	Y	Y	N	Y	Y	Y	Y	Y	90
3	Fambrini et al., 2013 [40]	Y	Y	Y	Y	Y	Y	Y	Y	Y	Y	100
4	Wu et al., 2005 [26]	Y	Y	Y	Y	Y	N	N	Y	Y	Y	80
		**Questions Assessing Cross-Sectional Studies**										
1	Ji et al., 2017 [23]	Y	Y	Y	Y	Y	Y	Y	Y			100

Y = Yes; N = No.

Case-control studies assessment

Questions:

1. Were the groups comparable other than the presence of disease in cases or the absence of disease in controls?
2. Were cases and controls matched appropriately?
3. Were the same criteria used for the identification of cases and controls?
4. Was exposure measured in a standard, valid and reliable way?
5. Was exposure measured in the same way for cases and controls?
6. Were confounding factors identified?
7. Were strategies to deal with confounding factors stated?
8. Were outcomes assessed in a standard, valid and reliable way for cases and controls?
9. Was the exposure period of interest long enough to be meaningful?
10. Was appropriate statistical analysis used?

Cross-sectional studies assessment

Questions:

1. Were the criteria for inclusion in the sample clearly defined?
2. Were the study subjects and the setting described in detail?
3. Was the exposure measured in a valid and reliable way?
4. Were objective, standard criteria used for the measurement of the condition?
5. Were confounding factors identified?
6. Were strategies to deal with confounding factors stated?
7. Were the outcomes measured in a valid and reliable way?
8. Was appropriate statistical analysis used?
